# Improving Eye-Tracking Data Quality: A Framework for Reproducible Evaluation of Detection Algorithms

**DOI:** 10.3390/s24092688

**Published:** 2024-04-24

**Authors:** Christopher Gundler, Matthias Temmen, Alessandro Gulberti, Monika Pötter-Nerger, Frank Ückert

**Affiliations:** 1 Institute for Applied Medical Informatics, University Medical Center Hamburg-Eppendorf, 20246 Hamburg, Germany; f.ueckert@uke.de; 2 EyeTrax GmbH & Co. KG, 49076 Osnabrück, Germany; matthias.temmen@eyetrax.de; 3 Department of Neurology, University Medical Center Hamburg-Eppendorf, 20246 Hamburg, Germany; a.gulberti@uke.de (A.G.); m.poetter-nerger@uke.de (M.P.-N.)

**Keywords:** eye-tracking, pupil detection algorithm, methodological framework, detection quality

## Abstract

High-quality eye-tracking data are crucial in behavioral sciences and medicine. Even with a solid understanding of the literature, selecting the most suitable algorithm for a specific research project poses a challenge. Empowering applied researchers to choose the best-fitting detector for their research needs is the primary contribution of this paper. We developed a framework to systematically assess and compare the effectiveness of 13 state-of-the-art algorithms through a unified application interface. Hence, we more than double the number of algorithms that are currently usable within a single software package and allow researchers to identify the best-suited algorithm for a given scientific setup. Our framework validation on retrospective data underscores its suitability for algorithm selection. Through a detailed and reproducible step-by-step workflow, we hope to contribute towards significantly improved data quality in scientific experiments.

## 1. Introduction

Eye-tracking technology has become pervasive in recording, assessing, and evaluating eye movements, gazes, and pupil reactions across diverse research fields [1]. The methodological approach gained increasing interest as a non-invasive, easy-applicable measure of brainstem activity in behavioral sciences and provides an indirect readout marker for brain state activity, alertness, and arousal levels [2]. In human and animal models, changes in attention, mental effort, and emotional distress are tracked by spontaneous fluctuations in pupil diameter [3,4,5]. Accordingly, pupillometry was used as an experimental measure to continuously monitor variations in wakefulness during periods of walking [6], a go/no-go signal detection task [6], and an auditory oddball detection task, where the pupil diameter exhibited a U-shaped relationship with the reaction time performing the task with shortest latencies at intermediate pupil diameters [7]. Thus, pupil diameter is a useful indicator of brain state, revealing neural mechanisms by which the brain is controlled on a moment-to-moment basis to predict optimal behavioral performance.

As the demand for objective measurements continues to grow, research and industry are driven to refine the required eye-tracking technology. Reliable measurements become even more important given their growing importance in medicine as an indirect biomarker to track disease states or to differentiate between disease entities. Pupil responses are used to discover subclinical affections of the optic nerve [8] and monitor fatigue in multiple sclerosis [9]. Quantitative pupil observations are widely used in intensive care units for monitoring patients with severe intracranial pathology [10] and are discussed to be useful in Alzheimer’s disease detection, diagnosis, and monitoring, alone or in combination with additional biomarkers [11]. In Parkinson’s disease, early pupillometry changes are observed even in preclinical stages [12], which are further modified depending on the disease stage throughout the disease [13]. Advancements in these fields, resulting in reduced costs, decreased complexities, and improved accuracy, are expected to further enhance the prevalence and significance of eye-tracking technology in the future [14].

Since the late 19th century, stationary eye trackers have been extensively researched for scientific purposes [15]. More recently, head-mounted video oculography has emerged as a valuable alternative, enabling experimental setups that were previously impossible [16]. Advancements in optical technology have led to the incorporation of cameras into glasses-like devices or virtual reality headsets, enabling continuous eye recording using non-visible near-infrared light. Unlike the proprietary devices used in the past, this technology offers transparency in the recording process and allows for re-evaluation with advances in analysis methods: affordable off-the-shelf technology is readily available or could be tailored to specific needs [17,18]. Consequently, this eye-tracking technology aligns directly with the four principal components of open science [19].

Despite the advantages of such flexible and archivable tailor-made solutions, a significant responsibility lies in fine-tuning the factors that influence the recording process. Alongside the physical camera setup, the pupil detection algorithms represent a crucial component of the eye-tracking pipeline. Despite the apparent simplicity of detecting the corresponding blob of pixels in a near-infrared image, this task remains challenging and unsolved. Factors such as contact lenses, dust, makeup, and other external influences further complicate the measurement process [20]. Given the plethora of available algorithms, each with distinct advantages and disadvantages, researchers must carefully choose a suitable method and associated hyperparameters based on their specific research objectives [21]. This publication aims to provide a framework for making such choices that are grounded in reliable evidence and ease of application.

Recording and analyzing eye-tracking data present challenges that are far from trivial [22]. However, these well-known complexities have resulted in a significant body of published knowledge and advice in literature [23,24]. These insights encompass a wide range of topics, including recording setups and devices [14,15], strategies for optimizing data quality [25], and proper analysis methods [26], providing valuable guidance for the appropriate use of this powerful methodology. As a result, researchers who aim to utilize eye-tracking technology can benefit from a rich ecosystem of scientific work that serves as a foundation for their investigations.

### 1.1. Navigating the Landscape of Eye-Tracking Algorithms

To assist applied researchers in selecting an appropriate pupil detection algorithm for their measurement pipeline, evaluation surveys like that of Fuhl et al. [27] have been conducted to compare and rate these algorithms. The diversity of potential recording designs has led to the existence of different datasets [28]. These datasets have been used for training or evaluating pupil detection algorithms, resulting in varying types of labels associated with the samples depending on three major types of algorithms:

1. The most fundamental type of annotation involves detecting the pupil center within the image [27]. With the two-dimensional coordinates of the detected pupil center and a commonly estimated three-dimensional eye model, subsequent procedures can calculate the corresponding eyeball rotation [29].
2. In scenarios where pupil size is of interest, such as pupillography, algorithms typically yield the best-fitting ellipse enclosing the pupil [17]. The output provides information on the size of the major and minor axes along with their rotation and two-dimensional position.
3. A more versatile representation utilizes a segmentation map covering the entire sample [28]. This segmentation mask is a binary mask where only the pupil is indicated. Some of these algorithms may also provide data on other eye components, such as the iris and sclera. Theoretically, this encoding allows for the use of partially hidden pupils due to eyelids or blinks.

Importantly, these three different annotation types create a hierarchy of information. The segmentation mask contains the most comprehensive information, but reducing it to an ellipse or even the pupil center is feasible. Researchers rely on large annotated datasets, assuming that algorithms performing well on them will generalize effectively to unseen setups and subjects. This generalization is crucial for practical applications where robustness and reliability are paramount considerations.

### 1.2. Choosing an Algorithm for Own Research

The concept of generalizability plays a crucial role in the assumption of algorithm performance [30]. However, assuming that sufficient performance on one dataset will translate to sufficient performance on a custom setup is not guaranteed. Several authors, such as Cazzato et al. [16], have emphasized the importance of custom adaptations to achieve satisfactory performance. These complexities arise from various aspects:

- Recorded samples vary significantly based on the camera position, resolution, and distance [31]. As a result, samples from different recording setups are not directly comparable. The non-linear transformation of the pupil when viewed from larger eye angles can present additional challenges to the algorithms [32].
- Algorithms often require the setting of hyperparameters that are dependent on the specific samples. Many of these hyperparameters have semantic meanings and are tailored to the camera’s position. While reusing published values may suffice if the setups are similar enough, obtaining more suitable detections will likely depend on fine-tuning these parameters.
- The population of subjects may differ considerably due to the context of the measurement and external factors. In medical contexts, specific correlated phenotypes may seriously hinder detection rates. There is a scarcity of published work, such as Kulkarni et al. [33], that systematically evaluates induced bias in pupil detection. Furthermore, challenges exist even within the general population, as documented by Fuhl et al. [27]. For instance, detecting pupils in participants wearing contact lenses requires detectors to perform well under this specific condition without introducing bias.
- Metrics used for performance evaluation can vary significantly between studies. Often, metrics are chosen to optimally assess a specific dataset or use case. For instance, the evaluation paper by Fuhl et al. [27] used a threshold of five pixels to classify the detection of the pupil center inside a sample as correct. While this choice is sound for the tested datasets, samples with significantly different resolutions due to another camera setup necessitate adopting alternative concepts.

Given these complexities, evaluating pupil detection algorithms requires careful consideration of their context. Making general claims of superior performance compared to all competitors is challenging. Consequently, custom considerations and evaluations in the application of pupil detection algorithms remain necessary to ensure their appropriate and reliable use in specific setups.

In addition to the hypothetical overall performance, various key concepts influence the decision in favor of one pupil detection algorithm over another. Similar to other machine learning use cases, softer concepts become relevant in the context of eye tracking [16]. For example, the transparency and interpretability of an algorithm might pose an important dimension, especially in sensitive medical areas where understanding the reasons behind a specific output becomes crucial for proper interpretation and validation. Another significant consideration is the licensing of the algorithm. Some algorithms may have licensing restrictions that limit their usage to non-commercial purposes. In academic settings, such licenses might be incompatible with classical open-source licenses like the GPL (General Public License), which requires re-licensing under the same terms. This can hinder the proper publication and dissemination required for transparent science. Taking all these factors into account further complicates the process of selecting an appropriate pupil detector beyond its assumed detection performance.

Even with a solid understanding of the literature, selecting the most suitable algorithm for a specific research project poses a challenge. To the best of our knowledge, no published piece of software provides more than six algorithms with the same API [17]. Therefore, empowering applied researchers to choose the best-fitting detector for their research needs is the primary contribution of this paper. Providing such a framework that facilitates the evaluation and comparison of pupil detection algorithms could allow researchers to make informed decisions and enhance the quality and reliability of eye-tracking data analysis.

## 2. Methods

The objective of this publication is the development and usage of a framework to empower researchers across diverse scientific disciplines to independently assess pupil detection algorithms in their experiments. The software must be inclusive, user-friendly, and not require in-depth knowledge of the algorithm’s technical details or implementation. Under the premise that the researcher should be able to optimize the measurements of their raw experimental data for the sake of more sustainable science.

### 2.1. Defining Criteria for the Framework

To uphold fundamental principles, we have defined essential criteria that any proposed framework must meet to effectively support academic practice. Automating the complexities associated with the evaluation process is paramount, minimizing the need for manual intervention. However, a precise definition of underlying constraints is necessary to ensure a sustainable and reusable utility.

- Flexibility: The proposed framework must exhibit maximum flexibility in its hardware and software environment for seamless execution. It should operate offline without reliance on remote servers, enabling widespread use in all countries.
- Accessibility: Additionally, the framework should not be tied to specific commercial systems that may pose accessibility issues due to license regulations or fees. Applied researchers should have the freedom to use the framework directly on their existing experimental systems, avoiding data duplication, preserving privacy, and simplifying knowledge management. As such, the framework should be compatible with a wide range of hardware, including UNIX-based operating systems commonly licensed as open-Source software, as well as the popular yet proprietary Microsoft Windows.
- Ease of setup: Once the system is available, setting up the framework should be straightforward and not require advanced technical knowledge. This may appear trivial but is complicated by the diversity of pupil detection algorithms. Existing implementations often depend on various programming languages and require multiple libraries and build tools, making the installation process challenging and time-consuming. To overcome this issue, the framework should not demand manual setup, but facilitating faster, and achieve assessment results easier.
- Scalability: The proposed framework must be scalable to handle the large volumes of samples in modern datasets, the diversity of algorithms, and the numerous tunable hyperparameters. Fortunately, the independence of algorithms and samples allows for easy parallelization of detections, enabling the efficient utilization of computational resources. The framework should be capable of benefiting from a single machine, multiple virtual machines, or even a cluster of physical devices, ensuring efficient exploration of the vast search space.
- Modularity and standardization: The framework should be designed with a modular approach and adhere to established standards and best practices. Embracing existing standards simplifies support and ensures sustainable development. Moreover, adhering to these standards allows for the re-use of individual components within the system, facilitating the integration of selected pupil detection algorithms into the final experiment seamlessly.
- Adaptability for researchers and developers: The framework should not only cater to researchers employing pupil detection algorithms but also be accessible to developers creating new detectors. By simplifying the evaluation process, developers may enhance their algorithms.

Given the defined constraints, a setup based on microservices and distributed computing appears highly appealing.

### 2.2. Inclusion Criteria of the Pupil Detection Algorithms

To ensure a comprehensive and meaningful comparison of pupil detection algorithms, we conducted a literature analysis to gather the currently available implementations. This investigation was built upon an extensive review by Zandi et al. [17], which served as the foundational reference for identifying relevant papers and follow-up publications. Additionally, the combination of considered algorithms based on their work and our additional research is shown in Table 1.

Please note that while this collection may not be fully exhaustive, it encompasses the majority of algorithms currently employed by researchers in the field. Each algorithm was individually evaluated for inclusion based on specific criteria:

- Availability of implementations: To ensure reproducibility, the published algorithms had to be accompanied by associated implementations. Although textual descriptions may exist, replicating an algorithm without access to its original implementation can introduce unintended variations, leading to inconsistent results. Therefore, only algorithms with readily available and accurate implementations as intended by the original authors were included.
- Independence of dependencies and programming languages: While no strict enforcement of specific dependencies or programming languages was imposed, a preference was given to algorithms that could be executed on UNIX-based systems. This choice was driven by the desire to avoid proprietary components and promote open-source software in science. As a result, algorithms solely available as compiled Microsoft Windows libraries without accompanying source codes were excluded. Similarly, algorithms implemented in scripting languages requiring a paid license, such as MATLAB, were not included.

The implementations that satisfied the criteria above were deemed suitable for inclusion within the framework. As part of our selection process, we did not require real-time execution of the algorithms, recognizing that offline analyses may be appropriate and necessary for specific experimental setups. To the best of our knowledge, this framework constitutes the most extensive collection available for evaluation purposes.

### 2.3. Architecture and Design of the Framework

**Figure 1:**
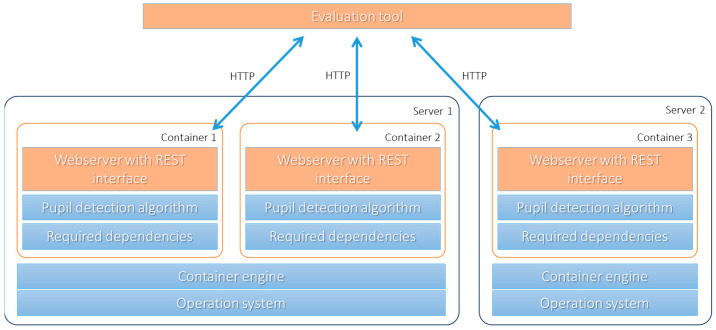
Visualization of a possible research setup involving two servers running three containers with pupil detection algorithms. All orange parts are part of the framework defined in this work. The evaluation tool sends the eye frames as images to the containers given their URL, where a web server providing a REST interface validates the data, hands it over to the pupil detection algorithm, and returns the value to the evaluation tool. As the number of containers per server is only limited in terms of the available computational resources, the system can scale flexibly.

The proposed, newly developed framework of this study employs a microservice architecture that was chosen for its inherent advantages in robustness, scalability, and compatibility (Figure 1). In this architecture, the minimal building blocks are represented as fully autonomous containers, providing self-contained and lightweight visualizations. These containers utilize the existing kernel of the operating systems, rendering complete simulations unnecessary, as seen in virtual machines. Each container encompasses all the essential dependencies required for the implementation of a specific pupil detection algorithm, encompassing the necessary software components, libraries, data dependencies, and the source code itself. This comprehensive encapsulation of the algorithm’s environment eliminates the need for manual installation and configuration, streamlining the deployment process for scientists.

The microservice architecture ensures the autonomy of each container, making them self-sufficient and isolated. As a result, the licensing of the container content corresponds precisely to the included code, enhancing intellectual property protection. This autonomy also contributes to the flexibility and suitability of the architecture in accommodating the diverse range of pupil detection algorithms.

Inspired by industry trends, the design of this architecture acknowledges the collective deployment advantages of autonomous services. Container runtimes, supporting various conditions and requirements, facilitate the execution of individual containers. The concept of hardware is largely abstracted in this setup, allowing the seamless utilization of physical hardware, virtual machines, or cloud-based resources. The versatility of this approach enables researchers to harness all available resources, facilitating scalability and enhancing the framework’s efficiency. As the experiment’s computational demands increase, the architecture scales nearly linearly with the available resources, promoting efficient utilization and resource allocation.

Services delivered in the form of containers rely on well-documented interfaces to facilitate interaction. The inner workings of the container implementation remain opaque and are treated as a black box. Therefore, communication and data transmission necessitate a robust network stack. We utilized the Hypertext Transfer Protocol (HTTP) due to its wide adoption and the availability of corresponding server and client libraries for most programming languages. Additionally, all the interfaces were designed to follow the Representational State Transfer (REST) style, which enables human-readable communication with the containers, enhancing compatibility and accessibility. To ensure standardization and interoperability, we employed the OpenAPI standard to allow for the automatic discovery, querying, and testing of the available resources. With these well-established building blocks, we designed a unified application interface for all pupil detectors, enabling their flexible use in diverse contexts.

Given the containers with well-defined REST application interfaces, most programming languages support sending samples and receiving estimated pupil data. Consequently, researchers can utilize a container runtime and develop evaluation procedures individually. However, managing the lifecycle of containers and handling input and output operations may become repetitive and cumbersome. To address this, the proposed framework offers an optional management tool designed for performance optimization. Controlled through a command line interface and a user-friendly configuration file, this software facilitates scalable and memory-efficient bulk processing of multiple samples. The obtained pupil estimates, encoded as JSON, then serve as the foundation for specific evaluations conducted by the researcher. This streamlined process simplifies the evaluation of multiple pupil detection algorithms, allowing researchers to focus on the core scientific aspects of their experiments.

### 2.4. Validation Data and Procedure

To demonstrate the potential of our proposed framework, we utilized a novel dataset. As a prototypical example of a specific research setup that might often occur in scientific reality, we utilized the collection of eye movement data within a virtual reality (VR) environment. The immersive nature of VR often introduces constraints in terms of the camera positioning and angle. For the exemplary utilization of our proposed framework, we utilized a novel dataset originally recorded by the company eyeTrax GmbH & Co. KG (Osnabrück, Germany). Before data collection, the participants provided informed consent, ensuring compliance with ethical guidelines for human subjects research. The dataset comprised manually extracted frames from the eye videos of 44 pseudonymized participants. The recording itself was based on cameras by the company Pupil Labs GmbH (Berlin, Germany) recording both eyes with a temporal resolution of 200 frames per second and a spatial resolution of 200 by 200 pixels. The secondary usage of this novel dataset ensured that it had not been previously used for training of machine-learning-based algorithms. Accordingly, it allowed us to analyze the data as a representative sample of a hypothetical population that could be observed within a specific research setup or commercial application.

In general, our framework is flexible enough to account for an unsupervised setup where, once a new pupil detection algorithm with unique advantages is developed, its authors may choose to test its consistency with existing algorithms. However, most authors will likely try to find the best approach for their setup. In this case, a labeled ground truth dataset is required. We performed manual annotation of the center of the pupil on four images per subject to establish a diverse gold standard. To ensure a representative sample, the 176 frames were randomly selected and stratified for both the left and right eye. In instances where the pupil was only partially visible, such as shortly before a blink, we approximated the center of the pupil and indicated the value as an approximation to understand the behavior of the algorithms under challenging conditions.

## 3. Results

The notion that a single pupil detection algorithm could universally cater to all scenarios is highly implausible. In this chapter, we describe an exemplary analysis conducted using our proposed framework to identify an optimal pupil detection algorithm for our given scientific setup. For the following steps, an interactive Jupyter Notebook is available to replicate the findings and apply them to custom data without advanced knowledge in a programming language.

### 3.1. Defining our Evaluation Criteria

Given the definition of our tasks as a supervised task and our labeled ground truth, we need to define our metric of interest. A possible option would be a binary label indicating whether an observation was similar to the specific ground truth.

**Figure 2:**
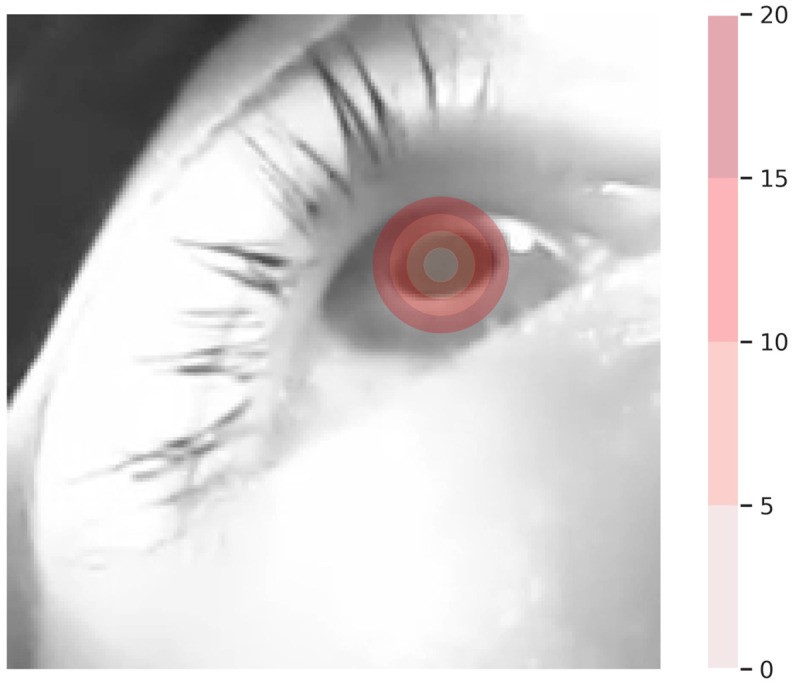
Visualization depicting the influence of diverse error magnitudes, quantified by the Euclidean distance between the annotated pupil center and potential predictions, on raw samples sized at 200 by 200 pixels. The center of the circles represents our ground truth annotation. Notably, errors surpassing 10 pixels appear substantial and may significantly compromise the accuracy of the detection process.

For our hypothetical setup, we choose to rather measure the error continuously as the Euclidean distance from our reference value. For easier interpretation of the findings, Figure 2 provides an overview of the significance of such continuous error measurements in our specific eye-tracking setup. That visualization helps us determine which values might be considered sufficient and which values might be considered good. Given the specific resolution and our human intuition, only an estimate below 10 pixels appears appropriate.

### 3.2. Generating the Predictions of all Pupil Detection Algorithms

Given the annotated ground truth, we can generate the predictions of the individual algorithms. For this purpose, we provide two different approaches: A Python script could serve as a foundation for developing custom pipelines. The script parses command line arguments and prints the results in a text file with comma-separated values. For those users with limited programming experience, we alternatively provide software parsing a config file where the necessary configuration could be defined with a simple text editor. While an additional graphical user interface may be even easier, we decided against its implementation due to the difficulties of implementing it in a platform-independent way. We applied all pupil detection algorithms with their default hyperparameters to the entire dataset.

### 3.3. Evaluation of the Pupil Detection Algorithms

**Figure 3:**
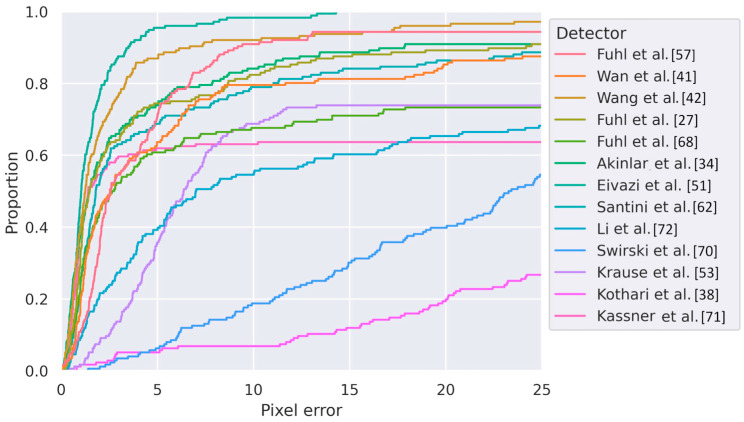
Empirical cumulative distribution functions illustrate the (Euclidean) pixel error across various pupil detectors, employing the default parameters as specified by the original authors on our dataset. The ordinate directly represents quantiles, with steeper curves indicating algorithms better suited to the specific dataset. Notably, certain algorithms predict over 80 percent of samples with errors below five pixels, while others encounter greater challenges. It is crucial to note that the algorithms were not fine-tuned, and the results should be interpreted as the lower boundary of achievable detection quality [27,34,38,41,42,51,53,57,62,68,70,71,72].

The pupil detection algorithms can be evaluated. Utilizing the annotated ground truth dataset and the predictions of the pupil detection algorithms, we calculated the Euclidian distance between both positions in pixel space to obtain the errors. These values are depicted in Figure 3 and serve as a quantitative measure of the accuracy and performance of each algorithm in capturing the true pupil center positions.

Upon initial examination, visualizing the errors using empirical cumulative distribution functions (ECDF), $f_{i}$ offers significant advantages for comparing the performance of different pupil detection algorithms. Firstly, by restricting the x-axis to the error values already visualized in Figure 2, we can estimate the number of samples for which individual detectors produce entirely incorrect results. The presence of outliers and large values does not hinder visualization as in a histogram [73].

Secondly, interesting quantiles like the median could be directly read from the y-axis. These robust estimators provide valuable insights into the quality of each algorithm’s performance. Finally, the individual functions of the ECDF offer a visual means of estimating the overall performance of each detector, similar to interpreting a receiver operating characteristic (ROC) curve. The steepest and most leftward rise to 100% corresponds to the most appropriate detector given the characteristics of the recorded samples, subjects, annotators, and evaluation metric. Accordingly, the detector by Eivazi et al. [51] appears to be a promising candidate for application on similar data.

### 3.4. Testing for Statistically Significant Performance Differences in Pupil Detectors

The empirical cumulative distribution functions serve as a basis for a first comparison of the pupil detection algorithms. However, it is essential to recognize that they are approximations of the true underlying distributions $F_{i}(x)$. To reason under the resulting uncertainty, estimates of confidence intervals using methods like the Dvoretzky–Kiefer–Wolfowitz inequality or statistical tests become necessary. As an exemplary research question, a researcher might aim to determine if utilizing a separate pupil detection algorithm is worth the effort compared to sticking with the existing algorithm by Pupil Labs, which is already in use. In this context, the researcher could define the null hypothesis ($H_{0}$) as “$F_{i}\left(x\right)\geq F_{j}\left(x\right)$ for all x,” and the alternative hypothesis ($H_{1}$) as “$F_{i}\left(x\right)<F_{j}\left(x\right)$ for all x.”

For comparing the empirical cumulative distribution functions, the Kolmogorov–Smirnov test provides a robust and non-parametric foundation for generating additional evidence. Setting $f_{i}$ to represent the best-performing algorithm and $F_{j}$ to represent the algorithm by Pupil Labs, the test rejects the null hypothesis with a *p*-value less than 0.0001. Consequently, we can safely assume that the alternative pupil detection algorithm might be more suitable for recording setups resembling the annotated ground truth. This finding further supports the utility of error visualization and statistical tests in making informed decisions regarding the choice of pupil detection algorithms for specific eye-tracking scenarios.

## 4. Discussion

The increasing popularity of eye-tracking technology for addressing sophisticated research questions has led to greater access to raw recordings, promoting reproducible research in response to the demand for open science. Consequently, there is a growing need for efficient tools to optimize the employed methods.

In this context, our developed framework addresses this requirement by providing building blocks that allow practitioners to easily assess the suitability of various algorithms for different research paradigms. The time-consuming manual setup of the requirements and environments for each approach is replaced by a solution providing clear interfaces. Its flexibility enables seamless integration with a wide range of published pupil-detection algorithms and eye-tracking setups. The standardized interface further facilitates the implementation of custom evaluation suites, the integration into existing software, and the inclusion of novel detectors into the framework.

However, certain limitations must be considered for practical application. Firstly, the currently available implementations may not be optimized for speed, prompting the exploration of optimized implementations, offloading work to remote servers, or employing parallel processing to enhance performance. It is important to emphasize that the framework is primarily intended for offline analysis after data recording, not real-time applications during experiments. Secondly, the containers used as building blocks for hardware independence are not virtual machines. Accordingly, they do not account for different processor architectures. If the pupil detectors or their dependencies rely on those subtle differences for increased performance, we cannot compensate. Running some of the detectors is not possible, for example, on those devices facilitating ARM chipsets. Thirdly, the retrospective nature of the analysis relies on access to raw data, making the recording of video streams necessary to ensure re-analysis and consistency in future studies. Ensuring access to raw data rather than solely derived values like pupil center and diameter remains crucial due to the wide range of devices and hardware configurations available. Finally, although our framework significantly reduces the workload for researchers in evaluating algorithms for their specific workflow, some manual labor is still required. Currently, the selection of algorithm parameters cannot be automated, necessitating researchers to examine these parameters manually for optimization purposes after an initial selection. Automation efforts could enhance the framework’s usability and efficiency.

For future work, we additionally suggest expanding the framework to include other essential pupil parameters beyond the pupil center, such as pupil ellipse measurements, which are crucial for pupillometry studies. Integrating these parameters would lead to a more comprehensive and accurate characterization of pupil behavior. Additionally, we propose employing the framework to investigate methodological questions beyond individual setups. For instance, it could facilitate a comparative analysis of various state-of-the-art algorithms for underrepresented groups, thus mitigating potential biases in eye-tracking studies.

## 5. Conclusions

The notion that a single pupil detection algorithm could universally handle all eye-tracking research scenarios is highly implausible. We present a comprehensive, flexible, scalable, easy-to-set-up, modular, and adaptable framework to identify the best-suited algorithm for a given scientific setup. While certain limitations exist, our software presents a versatile and effective tool for researchers seeking to enhance the accuracy and reliability of their eye-tracking analyses. In providing a unified interface for 13 pupil detection algorithms, we more than double the number of algorithms currently usable within a single software package. Given its representation of the currently available algorithms, we hope to further stimulate the methodological development in eye-tracking research.

## Figures and Tables

**Table 1:** Overview of publications featuring pupil detection algorithms examined in our study. A checkmark enclosed in brackets denotes cases where the code is unavailable, but a compiled library is provided. Explanations for the exclusion of certain algorithms from the subsequent analysis are explicitly detailed in the last column.

Publication	Code Available?	Programming Language	Included?
Akinlar et al. [34]	✓	Python	✓
Xiang et al. [35]	-		Excluded: Code not available
Bonteanu et al. [36]	-		Excluded: Code not available
Cai et al. [37]	-		Excluded: Code not available
Fuhl et al. [28]	-		Excluded: Code not available
Kothari et al. [38]	✓	Python	✓
Larumbe-Bergera et al. [39]	-		Excluded: Code not available
Shi et al. [40]	-		Excluded: Code not available
Wan et al. [41]	✓	Python	✓
Wang et al. [42]	✓	Python	✓
Bonteanu et al. [43]	-		Excluded: Code not available
Fuhl et al. [44]	-		Excluded: Code not available
Han et al. [45]	✓	Python	Excluded: No weights for the neural network
Manuri et al. [46]	-		Excluded: Code not available
Bonteanu et al. [47]	-		Excluded: Code not available
Bonteanu et al. [48]	-		Excluded: Code not available
Bonteanu et al. [49]	-		Excluded: Code not available
Bozomitu et al. [50]	-		Excluded: Code not available
Eivazi et al. [51]	✓	Python	✓
Han et al. [52]	-		Excluded: Code not available
Krause et al. [53]	✓	C++	✓
Miron et al. [54]	-		Excluded: Code not available
Yiu et al. [55]	✓	Python	Excluded: Unable to specify the container
Fuhl et al. [56]	(✓)	C++	Excluded: Binary library not available for Linux
Fuhl et al. [57]	(✓)	C++	✓
George et al. [58]	-		Excluded: Code not available
Li et al. [59]	-		Excluded: Code not available
Martinikorena et al. [60]	✓	MATLAB	Excluded: Requires proprietary interpreter
Santini et al. [61]	✓	C++	Excluded: Temporal extension of another algorithm
Santini et al. [62]	✓	C++	✓
Vera-Olmos et al. [63]	✓	Python	Excluded: Unable to specify the container
Fuhl et al. [64]	-		Excluded: Code not available
Topal et al. [65]	-		Excluded: Code not available
Vera-Olmos et al. [66]	-		Excluded: Code not available
Fuhl et al. [67]	-		Excluded: Code not available
Fuhl et al. [27]	✓	C++	✓
Fuhl et al. [68]	✓	C++	✓
Javadi et al. [69]	✓	.NET	Excluded: Not available for Linux
Świrski et al. [70]	✓	C++	✓
Kassner et al. [71]	✓	Python	✓
Li et al. [72]	✓	C++	✓

✓: Yes; (✓): Code is unavailable, but a compiled library is provided; -: No.

## Data Availability

The framework is available on GitHub https://github.com/Christopher22/ommatidia, accessed on 20 April 2024. The datasets of the individual predictions by the algorithms generated and analyzed during the current study required for replication are available in the ZFDM repository with the DOI https://doi.org/10.25592/uhhfdm.13720. The raw frames are not publicly available due to privacy constraints but are available from the corresponding author upon reasonable request.
